# Immunoglobulin Free Light Chains Are Increased in Hypersensitivity Pneumonitis and Idiopathic Pulmonary Fibrosis

**DOI:** 10.1371/journal.pone.0025392

**Published:** 2011-09-28

**Authors:** Tom Groot Kormelink, Annie Pardo, Karen Knipping, Ivette Buendía-Roldán, Carolina García-de-Alba, Bart R. Blokhuis, Moises Selman, Frank A. Redegeld

**Affiliations:** 1 Division of Pharmacology and Pathophysiology, Utrecht Institute for Pharmaceutical Sciences, Faculty of Science, Utrecht University, Utrecht, The Netherlands; 2 Faculty of Sciences, Universidad Nacional Autónoma de México, Mexico, México; 3 Danone Research - Centre for Specialised Nutrition, Wageningen, The Netherlands; 4 Instituto Nacional de Enfermedades Respiratorias “Ismael Cosío Villegas”, México DF, México; University of Medicine and Dentistry of New Jersey, United States of America

## Abstract

**Background:**

Idiopathic pulmonary fibrosis (IPF), a devastating lung disorder of unknown aetiology, and chronic hypersensitivity pneumonitis (HP), a disease provoked by an immunopathologic reaction to inhaled antigens, are two common interstitial lung diseases with uncertain pathogenic mechanisms. Previously, we have shown in other upper and lower airway diseases that immunoglobulin free light chains (FLCs) are increased and may be involved in initiating a local inflammation. In this study we explored if such a mechanism may also apply to HP and IPF.

**Methods:**

In this study we examined the presence of FLC in serum and BAL fluid from 21 IPF and 22 HP patients and controls. IgG, IgE and tryptase concentrations were measured in BAL fluid only. The presence of FLCs, plasma cells, B cells and mast cells in lung tissue of 3 HP and 3 IPF patients and 1 control was analyzed using immunohistochemistry.

**Results:**

FLC concentrations in serum and BAL fluid were increased in IPF and HP patients as compared to control subjects. IgG concentrations were only increased in HP patients, whereas IgE concentrations were comparable to controls in both patient groups. FLC-positive cells, B cells, plasma cells, and large numbers of activated mast cells were all detected in the lungs of HP and IPF patients, not in control lung.

**Conclusion:**

These results show that FLC concentrations are increased in serum and BAL fluid of IPF and HP patients and that FLCs are present within affected lung tissue. This suggests that FLCs may be involved in mediating pathology in both diseases.

## Introduction

Interstitial lung diseases (ILD) comprise a diverse group of disorders affecting the lung parenchyma that are classified together because they share similar clinical, radiographic, and physiologic features [1]. Two frequent and complex ILD are idiopathic pulmonary fibrosis (IPF) and hypersensitivity pneumonitis (HP).

IPF is a chronic fibrosing interstitial pneumonia of unknown aetiology limited to the lungs and associated with the histopathologic pattern of usual interstitial pneumonia (UIP) [2]. It is characterized by alveolar epithelial cell injury and activation, expansion of the fibroblast/myofibroblasts population forming the so called fibroblastic foci and the exaggerated accumulation of extracellular matrix [3], [4]. The disease is usually progressive and does not have effective therapy [5]. Hypersensitivity pneumonitis consists of a group of lung disorders resulting from exposure to a wide variety of organic particles causing an immunopathological reaction of the lungs in susceptible individuals [6]. One of the most frequent aetiologies of HP is the inhalation of bird-derived proteins that provoke the so-called pigeon breeders' disease (PBD). The clinical behavior is heterogeneous and may present as acute, sub-acute or chronic forms, often with overlap between these interrelated categories [7]. Importantly, patients with chronic HP may evolve to interstitial fibrosis, and in advanced stage may be very difficult to distinguish from IPF/UIP [8], [9]. Strong evidence indicates that sub-acute and chronic HP is primarily a T-cell mediated hypersensitivity [10]. Less is known about B lymphocyte involvement, although some participation is suggested by the antibody response to inhaled antigens resulting in high titers of circulating specific antibodies and the presence of plasma cells in the bronchoalveolar lavage mainly in sub-acute cases [11], [12].

Mast cell involvement in ILD pathology is uncertain but it is shown that increased numbers of mast cells are present in bronchoalveolar lavage (BAL) fluid of both IPF and HP patients [11], [13]–[17]. Moreover, these mast cells show activated phenotypes, the mast cell products histamine and tryptase are detectable in BAL fluid, and mast cell counts in lung biopsies positively correlate with the degree of fibrosis [15], [18]. Interestingly, mast cells can be rich sources of profibrotic cytokines, growth factors and proteases which are known to modulate the fibrotic process like transforming growth factor-β (TGF-β), IL-1, IL-4, IL-13, tumor necrosis factor-α (TNF-α), chymase, and tryptase [14], [19]–[21]. Furthermore, mast cells can produce a plethora of mediators involved in the recruitment and activation of other inflammatory cell types like lymphocytes and monocytes.

Previously we have shown that immunoglobulin free light chains (FLCs) can mediate antigen-specific mast cell activation [22]. FLC concentrations are increased in different immune disorders in which mast cells appear to play a prominent function like rheumatoid arthritis, inflammatory bowel disease, and multiple sclerosis, and some respiratory disorders like asthma and rhinitis [23]–[26]. The aim of this study was to investigate FLC expression in IPF and HP patients, and relate these findings to immunoglobulin concentrations, inflammatory cells present in affected lungs, and pulmonary function tests. Furthermore, the number of mast cells and its activation state was analyzed in both patient groups and compared to controls.

## Methods

### Study population

Blood and BAL samples were obtained from 21 patients with IPF and 22 patients with chronic HP induced by exposure to avian antigens (pigeon breeders' disease). None of the patients had been treated with corticosteroids or immunosuppressive drugs at the time of the study. As controls, blood samples and BAL fluids were achieved from 11 and 4 healthy individuals respectively. The study was approved by the Bioethics committee at the National Institute of Respiratory Diseases, and informed consent was obtained from all subjects.

Diagnosis of IPF was performed according to the American Thoracic Society/European Respiratory Society consensus [27]. Open lung biopsy was performed in 46% of the patients and all of them showed typical microscopic findings of usual interstitial pneumonia [28]. In the absence of biopsy, patients had to fulfil the criteria of the ATS/ERS international consensus, including a confident HRCT scan [29]. Diagnosis of chronic HP was obtained as described elsewhere [10] and based on the following criteria: a) history of pigeon exposure and positive serum antibodies against avian antigens; b) clinical, radiological, and functional features of an ILD with ≥6 months of symptoms; c) >30% lymphocytes in BAL fluid; and d) lung histology compatible with HP.

### Bronchoalveolar lavage

BAL was performed through flexible fiberoptic bronchoscopy under local anaesthesia as previously described [30]. Briefly, 200 ml of normal saline was instilled in 50-ml aliquots, with an average recovery of 60%–70%. The recovered BAL fluid was centrifuged at 250 g for 10 min at 4°C. The cell pellet was resuspended in 1 ml of phosphate buffered saline (PBS) and an aliquot was used to evaluate the total number of cells. Other aliquots were fixed in carbowax, stained with hematoxylin and eosin, and used for differential cell count. Supernatants were kept at −70°C until use.

### Total FLC, IgE, IgG, and tryptase measurement

Total serum or BAL fluid kappa (κ) and lambda (λ) FLC concentrations were determined using an ELISA adapted from Groot Kormelink *et al* [23]. In brief, plates were coated (o/n; 4°C) with goat anti-mouse IgG (M4280, Sigma, Zwijndrecht, The Netherlands) and subsequently blocked (1 hour; RT) and incubated with mouse anti- human kappa (κ) or lambda (λ) Ig-FLC MAb's (obtained from Dr. A. Solomon, Tennessee, US). After incubation with different dilutions of samples and standards (Binding Site, Birmingham, UK), plates were incubated with HRP-labelled goat F(ab′)_2_-anti human kappa or lambda immunoglobulin light chain antibodies (AHI1804 and AHI1904, respectively, Biosource, USA). TMB was used as a substrate. Per sample, at least three data points within the range of the standard curve were used to estimate the FLC concentration. For measurements of total IgE, total IgG and tryptase in BAL fluid the ImmunoCAP 100® system (Phadia AB, Uppsala, Sweden) was used. For total IgG, BAL samples were pre-diluted 100 times in specific IgA/IgG sample diluent. All tests were performed according to the manufacturer's instructions. Total IgE concentrations are expressed in kU/L, total IgG concentrations in mgA/L and tryptase concentrations in µg/L. Total IgE antibody concentrations ≥0.35 kU/L, total IgG antibody concentrations ≥0.07 mgA/L, and tryptase concentrations ≥1 ug/L were considered positive.

### Immunohistochemistry

Immunohistochemistry was performed on 4 µm thick formalin-fixed, paraffin embedded lung tissue sections from 5 IPF, 5 HP subjects and 3 controls. In short, serial tissue sections were subjected to heat induced antigen retrieval for 25 minutes (S1700, Dakocytomation, Haverlee, Belgium), blocked with PBS-T/3%BSA/3% normal goat serum for 1 hour, and incubated (over night) with the following primary antibodies diluted in blocking buffer: mouse-anti-human kappa FLC (Fκ-C8) and mouse-anti-human lambda FLC (Fλ-G9) (both obtained from Dr. A. Solomon, Tennessee, USA), mouse-anti-human CD138 (clone MI15, Dakocytomation, Haverlee, Belgium), rabbit-anti-human CD20 (clone BV11, Abcam,Cambridge, UK), and biotin-conjugated mouse-anti-human tryptase (G3361, Promega, Leiden, The Netherlands). Tissues were subsequently incubated with Alexa Fluor 568 goat-anti-mouse IgG, Alexa Fluor 488 goat-anti-rabbit IgG, or streptavidin Alexa Fluor 568 conjugate (all Invitrogen, Breda, The Netherlands) for 1 hour. Nuclei were counterstained with diamidino-phenylindole (DAPI, Invitrogen, Breda, The Netherlands). For mast cell number quantification, a mean number of mast cells per patient was calculated by averaging the number of mast cells counted in seven randomly taken pictures (200× magnification) per tissue. Sections were viewed with an Eclipse TE2000-U inverted microscope (Nikon) or a Zeiss LSM-510 confocal microscope. Images were analyzed using NIS elements BR 2.3 (Nikon) or Zen 2007 (Zeiss) software, respectively.

### Statistical analysis

FLC, IgG and IgE concentrations in serum and BAL fluid were compared using a One-Way ANOVA. Subsequently, a post-hoc Dunn's multiple comparison test was performed on all patients groups. Correlations between all serum and BAL fluid parameters were determined by Spearman correlation coefficient. Tissue mast cell numbers were analyzed using a One-Way ANOVA followed by a bonferroni multiple comparison test. P Values were considered significant when p<0.05. All analyses were performed using GraphPad Prism, version 4.03.

## Results

### Demographic and clinical features

Demographic and clinical features of the analyzed patient groups are shown in table 1. All patients exhibited clinical, radiologic, and functional evidence of ILD, with variable degrees of dyspnoea, decreased lung volumes, and hypoxemia at rest that worsened during exercise. Although the analyzed patient groups show considerable differences in age FLC concentrations are not much influenced by age in adults [31]–[33]. Furthermore, there is a difference in ratio of smokers/non-smokers between the groups but the 2 smoking controls had intermediate serum concentrations, suggesting that smoking does not significantly influence serum FLC.

**Table 1:** Demographic and clinical characteristics of the patients with hypersensitivity pneumonitis and idiopathic pulmonary fibrosis.

	*HP (n = 22)*	*IPF (n = 21)*	*Control (serum; n = 11)*	*Control (BAL; n = 4)*
Age, yr	52±12	66±8	42±20	53±15
Sex (M/F)	4/18	14/7	2/9	3/1
Current or former smoking	3/22	11/21	2/9*	1/4
**Bronchoalveolar Lavage**				
Macrophages, %	48±19	84±10		
Lymphocytes, %	50±19	12±7		
Neutrophils , %	0.8±0.9	2.4±3.4		
Eosinophils, %	1.2±2	1.8±2.9		
**Pulmonary Function Tests**				
DLCO, % predicted	46±20	42±18		
FVC, % predicted	61±16	63±24		
FEV1, % predicted	64±18	68±26		
SpO2, % at rest	88±4	87±5		
SpO2, % during exercise	75±19	81±6		
PaO2 mm Hg**	54.8±7.2	56.2±9		
ePSAP mm Hg	31±18	48±18		

Mean values are shown ± standard deviation.

* = unknown for 2 controls;

**PaO2 = arterial pressure oxygen; normal values at Mexico City altitude are 67±3 mmHg. SpO2 = pulse oxygen saturation. FEV1 = volume of air expired during the first second. FVC = forced vital capacity. DLCO = carbon dioxide diffusing capacity. ePSAP = estimated systolic pulmonary artery pressure.

### Increased serum and BAL fluid FLC concentrations in IPF and HP patients

**Figure 1:**
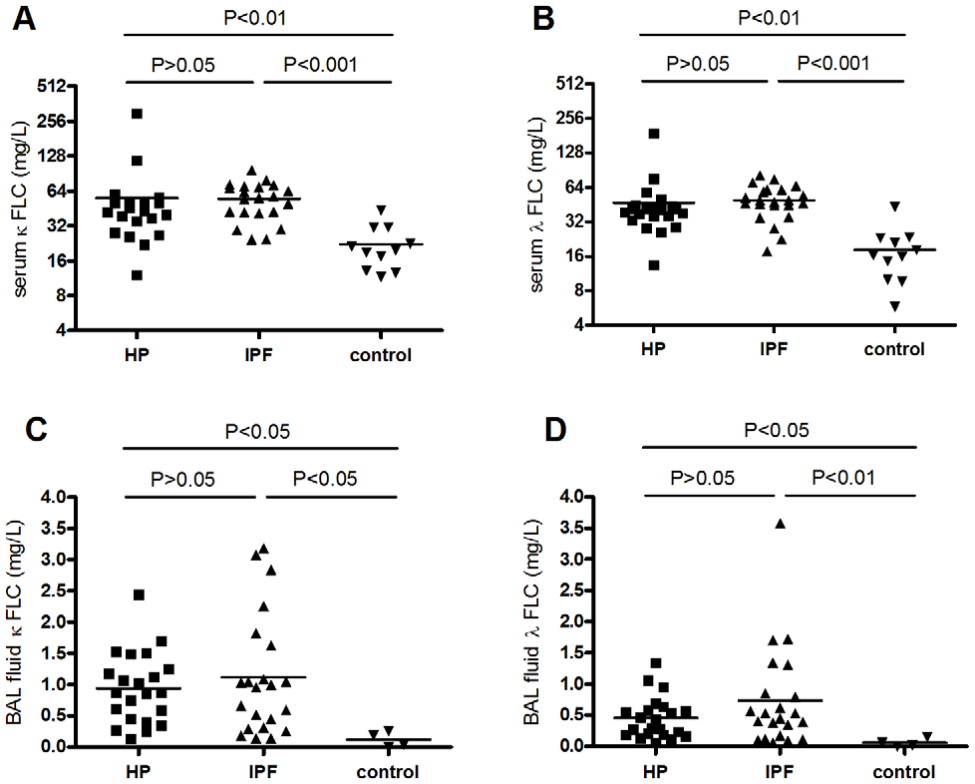
Free light chain concentrations in serum and BAL fluid from HP and IPF patients. Kappa and lambda FLC concentrations are highly increased in serum (**A** and **B**, respectively) and BAL fluid (**C** and **D**, respectively) of the HP and IPF groups compared with control subjects. Mean FLC concentrations are similar in the IPF and HP groups.

In serum and BAL fluid, FLC concentrations are significantly increased in HP and IPF patients as compared to healthy controls. Mean (±SEM) κ-FLC serum concentrations were 55.9±12.32 mg/L in HP, 54.7±4.04 mg/L in IPF, and 22.1±2.93 mg/L in control subjects (Figure 1A). For λ-FLC serum concentrations were 46.8±7.29 mg/L in HP, 46.6±3.44 mg/L in IPF and 18.3±3.02 mg/L in the control group (Figure 1B). Mean (±SEM) FLC concentrations in BAL fluid were 0.94±0.12, 1.11±0.20, 0.12±0.060 mg/L (κ-FLC) and 0.45±0.07, 0.73±0.17, 0.06±0.03 mg/L (λ-FLC) in the HP, IPF and control group, respectively (Figure 1C and D). FLC concentrations in BAL fluid showed no significant correlation with those in serum.

### Total IgG, IgE and tryptase concentrations in BAL fluid from HP and IPF patients

**Figure 2:**
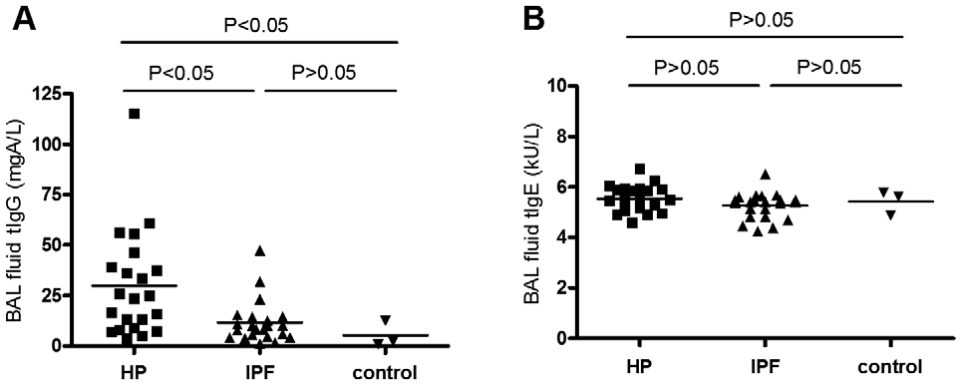
IgG and IgE concentrations in BAL fluid from HP and IPF patients. Total IgG concentration in BAL fluid was increased in the HP but not in the IPF and control group (**A**). Total IgE concentrations in BAL fluid were similar in the HP, IPF, and control group (**B**).

Both IgG and IgE antibodies were detectable in BAL fluid from HP and IPF patients and control subjects. The mean (±SEM) total IgG concentration in HP patients (29.8±5.58 mgA/L) was significantly higher than that in IPF patients (11.6±2.28 mgA/L) and control subjects (5.3±3.70 mgA/L) (both p<0.05). Total IgG concentrations in BAL fluid from IPF patients and controls did not differ significantly (Figure 2A). Total IgE concentrations were similar in all groups (Figure 2B). Tryptase was only detectable in 2 patients, one HP patient (9.27 µg/L) and one IPF patient (2.27 µg/L) (data not shown).

### Correlations between BAL fluid humoral factors and clinical parameters

In the HP and IPF patient groups we investigated the relationship between FLC and BAL fluid IgG concentrations and inflammatory cell numbers, and the clinical parameters as described in table 1. For FLC, a positive correlation was found between λ-FLC and DLCO% in the HP patient group (p = 0.0021; r = 0.62). A positive correlation was also found between total IgG concentrations and the number of BAL fluid lymphocytes in HP (p = 0.006; r = 0.57) and IPF (p = 0.037; r = 0.46) patients. A significant positive correlation between IgG and kappa and lambda FLC was found in IPF patients (IgG vs κ-FLC: p = 0.009; r = 0.55; IgG vs λ-FLC: p = 0.045; r = 0.44).

### FLC-positive cells, B cells, plasma cells and mast cells in lung tissue from HP, IPF and control subjects

**Figure 3:**
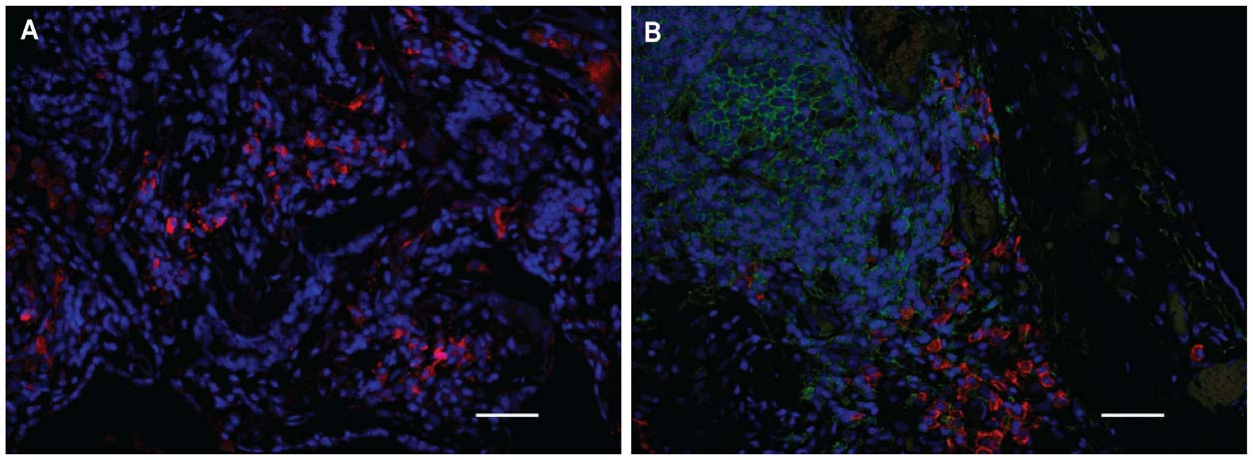
FLC-positive cells, B cells, and plasma cells in the lungs of HP and IPF subjects. Representative pictures are shown of numerous FLC-positive cells (both kappa and lambda FLC are stained red) (**A**), and B cells (green) and plasma cells (red) (**B**) which are found in HP and IPF lung tissue. Scale bar: 50 µm.

**Figure 4:**
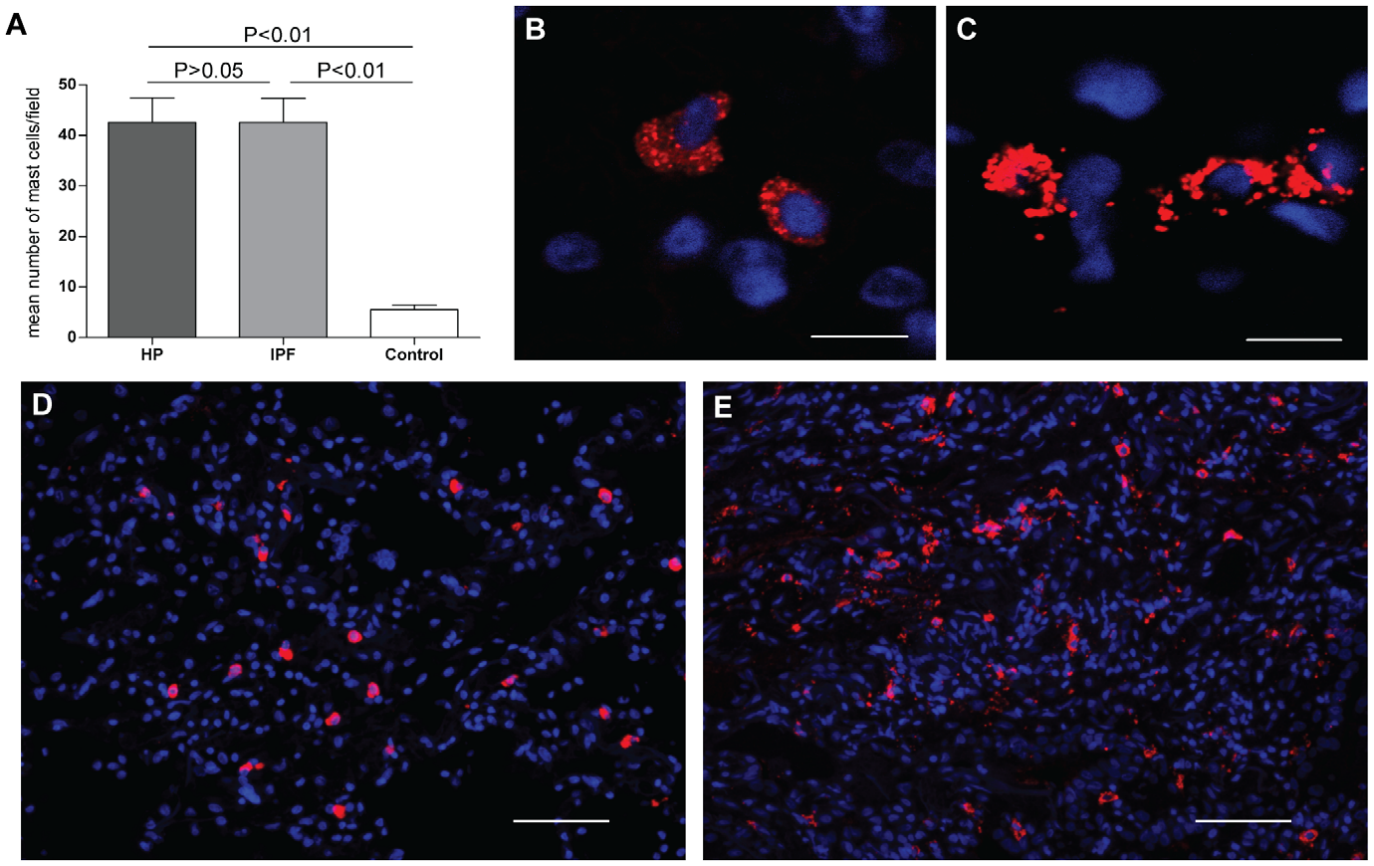
Numerous mast cells are found in the lungs of HP, IPF and control subjects. The number of mast cells is significantly higher in HP and IPF patients compared to controls (**A**). Per patient, the mean number of mast cells is calculated by averaging the number of cells counted in seven randomly taken pictures of the tissue. Bars represent the means ± SEM for 5 IPF and 4 HP patients and 3 controls. Representative pictures are shown of tryptase positive mast cells (red) with a non-activated phenotype as mainly found in control tissue (**B** and **D**), and tryptase positive mast cells with an activated/degranulated phenotype as mainly found in HP and IPF tissue (**C** and **E**). **B** and **C** are representative confocal images taken from tissue shown in **D** and **E**, respectively. Scale bar **B,C**: 10 µm; **D,E**: 50 µm.

Immunohistochemical analysis of lung tissue from all HP and IPF patients revealed similar staining patterns for FLC, CD20 (B-cells), CD138 (plasma cells), and tryptase (mast cells). Many FLC-positive cells were detected throughout the tissue as isolated cells or in small groups (Figure 3A). Numerous B cells and plasma cells were detectable, either as single cells or clustered in groups and in vicinity of each other (Figure 3B). B cells, and plasma cells were not detectable in control lung tissues, and FLC-positive cells were here only detected scarcely. Tryptase positive cells were present throughout all lung tissues, including control tissue. However, the number and density of mast cells was lower in the control tissue (Figure 4A) and morphological differences were dramatically apparent between mast cells in HP and IPF lungs as compared to mast cells in healthy control lungs (Figure 4B–E). Mast cells in control tissue had a non-activated phenotype (Figure 4B and D), whereas many mast cells in HP and IPF lungs showed signs of activation and degranulation (Figure 4C and E).

## Discussion

In the present study, we show that FLCs are increased in serum and BAL fluid from IPF and HP patients as compared to controls. Furthermore, FLC-positive cells are present in the lungs of IPF and HP patients. Total IgG concentrations were only increased in HP patients, whereas total IgE concentrations in IPF and HP patients were not different from healthy controls.

The pathological mechanisms involved in IPF and HP are yet unclear, but the putative contribution of mast cells in mediating fibrosis in both disorders has been recognized [15], [18], [34]. The high number of mast cells and their activated appearance in the lungs of IPF and HP patients compared to normal lung tissue observed in this study supports previous observations indicating mast cell involvement in both disorders. Interestingly, increased tissue or BAL fluid mast cell numbers were shown to correlate with the degree of fibrosis and activity of disease [11], [16], [17], [35]. Moreover, tryptase was shown to be present in BAL fluid from IPF and HP patients, supporting the morphological features of mast cell activation [18]. In this study however, we only found detectable concentrations of tryptase in BAL fluid from 2 of 44 patients. Because histological analysis of mast cells in lung tissue from IPF and HP patients clearly demonstrated their activated appearance, timing and location of sampling and/or analytical detection limits may explain the discrepancy with the BAL tryptase analysis.

Despite the activated mast cell appearance, a possible activation mechanism in IPF and HP lungs is not elucidated. The involvement of IgE in IPF and HP is not likely, because IgE concentrations in BAL fluid are not increased compared to healthy controls. Many other stimuli such as FLC, IgG, complement factors, toll-like receptor ligands, and bacterial and viral products can induce mast cell activation [22], [36]. In agreement with other studies, we found increased concentrations of total IgG in BAL fluid from HP patients and specific IgG against the sensitizing antigen have been demonstrated in HP lungs [12]. This suggests that IgG could be involved in triggering mast cell activation in lungs of HP patients. Alternatively FLCs, which we found significantly increased, could mediate antigen-specific mast cell activation [22]. Thus, together, FLCs and IgG might provide a mechanism by which antigen-specific mast cell activation takes place in the lungs of HP patients. In our previous work we have demonstrated a crucial role of FLC in experimental models for allergen-induced contact hypersensitivity, asthma, food allergy and IBD [22], [24], [25]. Whether antigen specific FLC can be detected in human body fluids is the subject of current research.

IPF is not an antigen-specific mediated disease, but viral infections seem to be common in these patients [37], [38] and although IPF is a multi-factorial disease, a growing body of evidence implicates viruses as co-factors, either as initiating or exacerbating agents [39]. Viral infections increase the occurrence of FLCs and interestingly, we have recently shown that FLC concentrations are greatly increased during viral myocarditis in mice and that FLC may play a protective role in the pathogenesis of disease [40].

The presence of FLCs in BAL fluid together with the high number of B cells and plasma cells in lung tissue in most IPF and HP patients supports the concept of local production of immunoglobulins in the lungs as suggested earlier for HP patients [11]. In IPF, primarily in areas of honeycombing changes (advanced disease), small lymphoid aggregates formed by B and T cells are usually noticed [41]. Likewise, B cells and plasma cells are seen within the lumen of bronchioles and alveolar walls [12]. The fact that FLC concentrations in serum are also increased might be caused by an overspill of locally produced FLC into the circulation. For example, such systemic increases in FLC have also been observed in asthma patients [25]. The low FLC concentrations detected in BAL fluid from controls are in line with the absence of plasma cells in healthy lung parenchyma [42].

FLCs and IgG concentrations appear not to be prognostic biomarkers for lung function capacity, since no clear correlations were found with different physiological and functional parameters. This lack of correlation impedes a conclusion on a functional role of FLCs in disease pathology, which could be due to the low number of patients per disease group available for our analysis. Furthermore, patients were in a medium/late stage of disease and this late phase in disease progression might hamper the detection of a functional role of FLCs in the initiation phase of disease. In addition, the effect of therapeutic treatment on the expression of FLCs is unexplored.

In conclusion, this study is the first to show the increased presence of FLCs in serum and bronchoalveolar lavage fluids from IPF and HP patients. Furthermore, in lung tissue from patients of both types of disease, many B cells, plasma cells, and activated mast cells can be found. These findings suggest that possible common immunologic mechanisms may be involved in both diseases, despite the differences in pathogenesis.

In former studies we described that antigen-specific FLC-mediated mast cell activation can regulate inflammatory responses in mice. This study shows presence of FLCs in HP and IPF, but whether they contribute to disease pathology via mediating mast cell activation remains presently unknown. Future research should also disclose if cell types other than mast cells known to be important in IPF and HP, like fibroblasts, macrophages, and neutrophils, are affected by FLC and whether prevention of FLC-induced activation of immune cells could be of therapeutic value in the treatment of HP and IPF.
